# Early post-transplant recurrence of ANCA vasculitis while on belatacept maintenance immunosuppression

**DOI:** 10.1007/s40620-022-01556-x

**Published:** 2023-01-04

**Authors:** Amogh Agrawal, Grace Yun Chong, Mary Elizabeth Fidler, Carl Henry Cramer II, Hatem Amer, Andrew John Bentall

**Affiliations:** 1 Division of Nephrology and Hypertension, Department of Medicine, Mayo Clinic, Rochester, MN USA; 2 The William J. von Liebig Center for Transplantation and Clinical Regeneration, Mayo Clinic, Rochester, MN USA; 3 Division of Anatomic Pathology, Department of Laboratory Medicine, Mayo Clinic, Rochester, MN USA; 4 Division of Pediatric Nephrology, Department of Pediatric and Adolescent Medicine, Mayo Clinic, Rochester, MN USA

**Keywords:** Kidney transplant, ANCA vasculitis, Recurrent disease, Immunosuppression, Belatacept

## Abstract

Post-transplant recurrence of ANCA-associated vasculitis (AAV) is infrequent, with recurrence within weeks of transplantation being even rarer. We describe an unusual case of AAV recurrence within 2 weeks post-transplant. Our patient received a deceased donor kidney transplant (KDPI 60%) after 6 years on hemodialysis for end-stage renal disease from AAV. She was induced with thymoglobulin and steroids, and maintained on belatacept, mycophenolate and prednisone. Time-zero biopsy showed acute tubular injury. Due to persistent delayed graft function by post-operative day 14, she underwent repeat biopsy, which showed focal segmental necrotizing and crescentic glomerulonephritis, with positive MPO, PR3 and negative anti-glomerular basement membrane antibodies. As her findings were in keeping with recurrent AAV, she underwent induction with rituximab, prednisone and intravenous immunoglobulin, with repeat rituximab 14 days later because of increasing B-lymphocyte counts. Belatacept was replaced with tacrolimus due to concerns with autoimmunity. Fortunately, renal function began to recover 4 days after treatment. In addition to highlighting potential immunologic mechanisms in AAV and the use of rituximab in post-transplant recurrence, our case suggests that for systemic autoimmune disease, patients maintained on belatacept must be monitored closely for recurrence, particularly in the setting of delayed graft function.

**Graphical Abstract:**

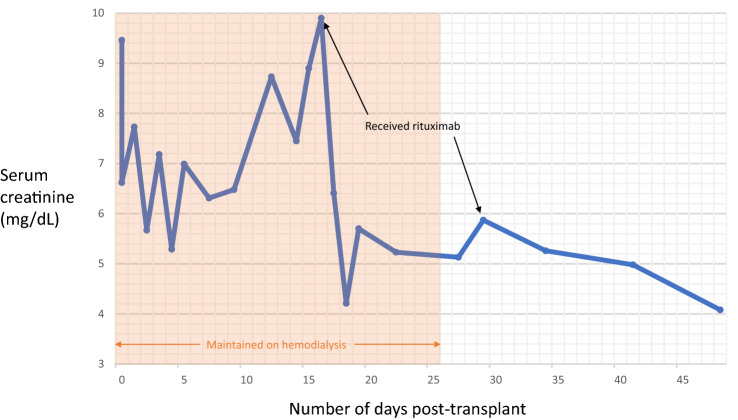

## Introduction

Post-kidney transplant recurrence of pauci-immune anti-neutrophil cytoplasmic antibody-associated glomerulonephritis (AAV) is estimated to be 10% in the current era of tacrolimus-based immunosuppression [1], with recurrence within weeks post-transplant being rare [2]. We describe an unusual case of AAV that occurred within 2 weeks post-transplant, while on a calcineurin-sparing maintenance immunosuppressive regimen of belatacept, mycophenolate mofetil and corticosteroids.

## Case description

A 56-year-old woman was diagnosed with AAV at age 15 after presenting with an orbital granuloma, initially treated by resection. She later presented with another orbital granuloma, then treated with cyclophosphamide and high-dose corticosteroids, with successful remission. Despite maintenance with azathioprine, she progressed to end-stage kidney disease, requiring hemodialysis at age 50.

Three months prior to transplant, her anti-myeloperoxidase (MPO) and proteinase 3 (PR3) antibody levels were 6.1 and 2.3, respectively. She was asymptomatic, deemed in remission by Rheumatology and recommended to continue her long-standing azathioprine.

She received a 5/6 HLA mismatched deceased donor kidney transplant (Kidney Donor Profile Index (KDPI) 60%) after 6 years on hemodialysis. The flow crossmatch was negative and there were no current or historic donor specific HLA antibodies. Cold-ischemia time was 19 h. Induction per protocol of patients under 65 years old consisted of thymoglobulin (2 mg/kg on post-operative days 0, 1, and 2) and methylprednisone. Maintenance immunosuppression consisted of belatacept, initiated per standard recommendations (initiation phase 10 mg/kg on post-operative days 0, 4, 14, and 28), mycophenolate mofetil (750 mg every 12 h), and prednisone (5 mg daily). The time-zero allograft biopsy showed acute tubular injury.

**Fig. 1:**
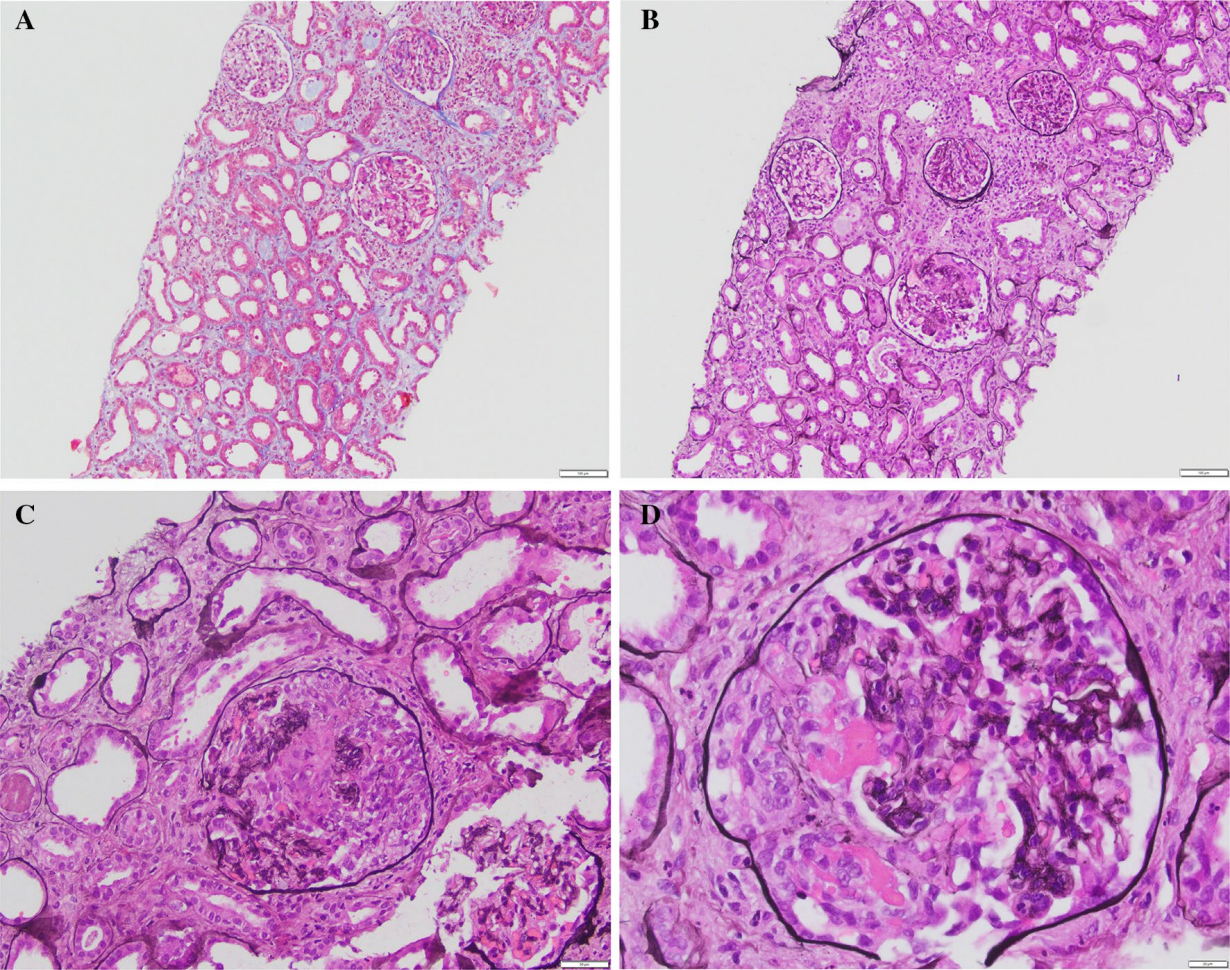
Histology demonstrates acute tubular injury with preserved glomeruli without significant chronic damage (**A**, 10 ×, Trichrome stain), however other glomeruli demonstrate crescentic changes on (**B**, 10 ×, Trichrome stain). **C** Demonstrates higher definition of the crescentic changes (20 ×, Jones stain), and on 40 × magnification fibrinoid necrosis and crescents are seen (**D**, 40 ×, Jones stain). Overall Banff score for the biopsy was g0, i1, t1, v0, ah0; cg0, ci1, ct1, ti3, cv1, mm0, ptc0. There was no IgG staining on immunofluorescence to suggest anti-GBM disease

Due to delayed graft function at post-operative day 14, she underwent allograft biopsy, which showed focal segmental necrotizing and crescentic glomerulonephritis in 9 of 21 glomeruli (Fig. 1A–D). Serologic work up included anti-MPO (4.4 U, negative < 0.4), anti-PR3 (1.1 U, negative < 0.4), negative anti-glomerular basement membrane antibodies and elevated CRP of 21.6 mg/L. Her findings were in keeping with early recurrent AAV.

She was induced with rituximab 1000 mg, prednisone 80 mg po daily and 2 doses of intravenous immunoglobulin (IVIg, 0.5 g/kg). She received a repeat dose of rituximab 14 days later due to residual B-lymphocyte counts. Belatacept was replaced with tacrolimus due to concerns that either its mode of action, or lack of a CNI had contributed to the early recurrence.

**Fig. 2:**
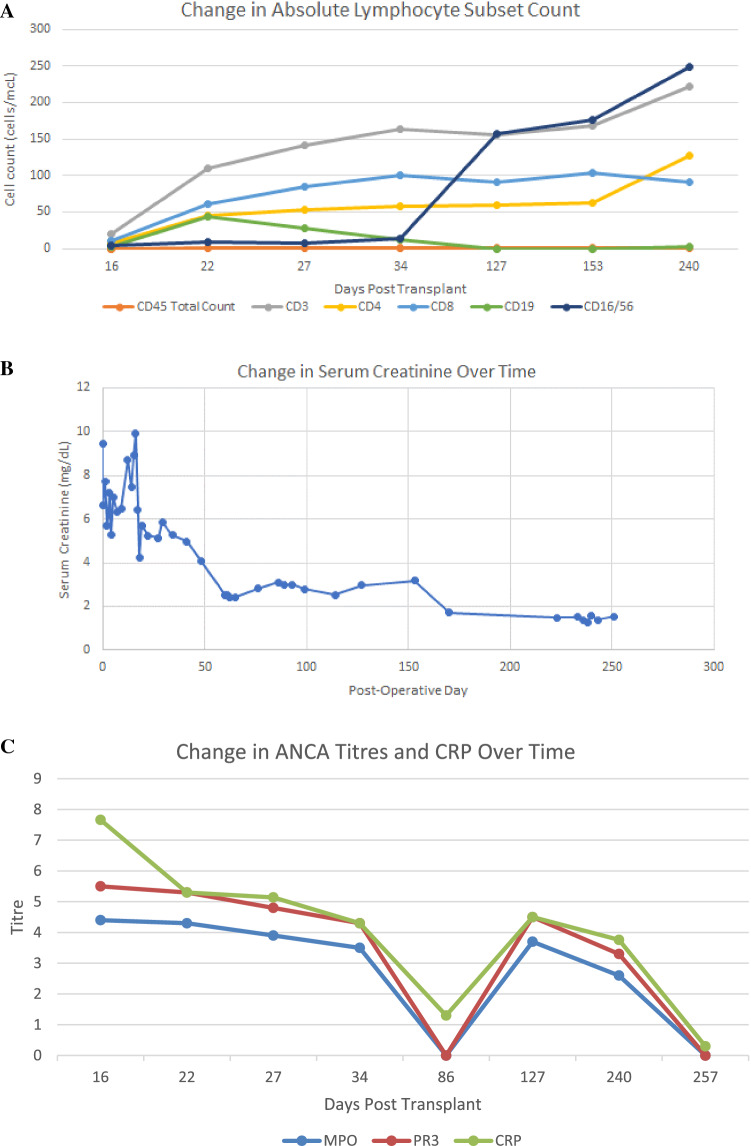
Serological and Lymphocyte changes with therapy. **A** Shows that changes in lymphocytes demonstrate persistent non-detectable levels of CD19 B lymphocytes but slow reconstitution of T cell components. **B** Shows the improvement in creatinine over time, relative to rituximab therapy. **C** Shows the modest reduction in MPO titer, which is similar to the changes in C-reactive protein (CRP). MPO and PR3 measures in units and CRP in mg/dL

The peripheral blood lymphocyte phenotype reflected induction with thymoglobulin with low T-cells (Fig. 2A). However, the proportion of memory T-cells was elevated (CD4 + CD45RO + memory T-cells – 94% CD4 cells, CD4 + CD62L + CD27 + CD45RO + – 49% CD4 cells, as well as CD8 + HLA DR + CD28 + T-cells – 78.7% of CD8 cells).

She began to produce urine four days after starting treatment with prednisone and rituximab, last requiring dialysis 11 days after the first rituximab infusion. Her creatinine continued to decrease thereafter, with serial measurements of her B-lymphocyte counts, MPO and PR3 titers also showing response to therapy (Fig. 2B and C). Follow-up biopsy on day 114 post-transplant showed fewer crescentic lesions (1 in 13 glomeruli) for which she received methylprednisone pulse, with no detectable CD19 cells in her serum. Kidney function continued to improve with current baseline creatinine of 1.5 mg/dL and eGFR of 36 mL/min/1.73m^2^.

Despite prophylactic doses of sulfamethoxazole/trimethoprim, she developed a Nocardia pulmonary infection 5 months post-transplant, requiring treatment with high dose sulfamethoxazole/trimethoprim. Other complications of her immunosuppression included CMV and BK viremia, which resolved with cessation of mycophenolate mofetil.

Her 1-year surveillance biopsy showed no signs of ongoing AAV with no immunoglobulin binding on immunofluorescence. She is now maintained on lower dose tacrolimus (target trough 4–6 ng/mL due to her infections) and prednisone.

## Discussion

The factors implicated in the immunopathophysiology of AAV are thought to cause defective degradation of neutrophil extracellular traps (NETs), allowing antigen-presenting cells of susceptible individuals to prime antibody-mediated responses against primarily MPO and PR3. When a pro-inflammatory milieu, triggered by an event such as infection, stimulates neutrophils to express MPO and PR3, these antibodies bind, activating neighboring neutrophils, causing excessive NET formation and subsequent endothelial cell damage. Post-transplant ischemia–reperfusion injury, almost universal in kidney transplants, has been shown to involve neutrophils, whose activation increases NET formation [3]. Though inflammation from ischemia–reperfusion injury may have triggered neutrophil activation in our patient, the cold ischemia time and preservation of the organ that this patient received was not an outlier compared to US averages for deceased donor kidney transplants. Moreover, given how rare early AAV recurrence is, ischemia–reperfusion injury is an unlikely trigger.

This case raises an interesting question. Did the atypical early recurrence of AAV result from the absence of tacrolimus or from a permissive effect of co-stimulatory blockade with belatacept? Induction with anti-thymocyte globulin and maintenance with tacrolimus have been postulated to reduce recurrence rates over time, by inhibition of T helper cells and subsequently, reduction of ANCA antibody production [1]. Other evidence supporting the efficacy of tacrolimus in maintaining remission is derived from studies showing lower remission rates in kidney transplant recipients when compared to dialysis patients [4], and indirect comparisons to higher relapse rates in native kidneys [5]. A lack of tacrolimus-based immunosuppression may have contributed, though it remains uncharacteristically rapid, even when compared to relapse rates in patients on cyclosporine-based regimens [6].

Belatacept prevents T-cell activation by competitively inhibiting the co-stimulatory signal between CD28 and its cognate receptors, CD80 and CD86. Its downstream effects can be gleaned from abatacept, its parent compound, which was shown to prevent differentiation of T-cells into T follicular helper cells, thereby suppressing humoral activation. Belatacept was shown to have superior renal function and similar 12-month patient and graft survival to cyclosporine [7]. However, trial data also showed increased acute cellular rejection within the first-year post-transplant [8], with other reports linking CTLA-4 inhibitors with new onset psoriasis [9] and chilblain lupus [10]. Though the exact mechanisms behind these pro-inflammatory phenomena remain unknown, several theories may explain the reactivation of AAV in our patient, and formed the basis for conversion to tacrolimus, without subsequent AAV recurrence.

One explanation for our patient’s abrupt reactivation of AAV is incomplete T-helper cell inhibition. Abatacept was known to have significantly less avidity for CD86 than for CD80 [11], with, in vivo studies showing poor activity in non-human primate transplant models [12]. Belatacept was designed to have significantly more avidity for both CD80 and CD86, though it still varies in its degree of binding [12]. This difference in binding may allow some antigen-presenting cells to deliver the co-stimulatory signal needed for T-helper cell activation. Further, mechanistic studies in ANCA-associated vasculitis identified CD28null T cells acting independently of the CD28/CD80 pathway, which may explain why CD80/CD86 inhibition in ANCA disease may not be optimal [13].

Memory T-cells may also have a role. Belatacept blocks CD28 in naïve T-cells, but not in memory cells [14]. Further, memory T-cells have been shown to express less CD28 than naïve ones, making them less dependent on co-stimulatory pathways for activation [15]. This is consistent with our finding of a significant proportion of remaining T-cells having a memory phenotype. Incomplete T-helper cell inhibition from belatacept may have allowed escape variants capable of activating MPO and PR3-specific B-cells.

Another possibility is through decreased T-regulatory cell function. T-regulatory cell activation requires engagement of surface-expressed CTLA-4, an interaction which belatacept competitively inhibits [15]. Further, thymoglobulin induction may have reduced the T-regulatory cells, allowing T-memory cells to proliferate [16].

Given the presence of ANCA antibodies and B-cells, we elected to treat with rituximab, and used IVIg for immunomodulation and protection from infectious diseases. Our patient’s subsequent biopsy demonstrated fewer crescents, suggesting that the active pathological process was responding to treatment. Indeed, the renal lesions were quiescent 1-year post-transplant.

We describe an unusually early post-transplant recurrence of AAV while on maintenance immunosuppression on a CNI-free regimen with belatacept. Though it responded successfully to rituximab and conversion to tacrolimus, our case suggests that the natural history of systemic autoimmune disease may differ from the experience with more traditional immunosuppression. It also highlights the burden of opportunistic infections triggered by intensive induction immunosuppression. Thus, patients maintained on belatacept must be monitored closely for recurrence, particularly in the setting of delayed graft function.
